# Myoclonus From Intoxication by Bismuth Iodoform Paraffin Paste (BIPP) Nasopharyngeal Packing

**DOI:** 10.7759/cureus.18530

**Published:** 2021-10-06

**Authors:** Rong Tan, Shermyn Neo, Jereme Gan, Ernest Fu, Ming Yann Lim, Hao Li

**Affiliations:** 1 Department of Otorhinolaryngology, Tan Tock Seng Hospital, Singapore, SGP; 2 Department of Neurology, National Neuroscience Institute, Singapore, SGP

**Keywords:** bipp, bismuth, iodoform, iodine, wound packing, nasopharyngectomy, myoclonus, toxicity, encephalopathy, video

## Abstract

Bismuth iodoform paraffin paste (BIPP) gauze is widely used as an antiseptic wound packing in otolaryngology, head, and neck surgery. Uncommonly, BIPP can cause intoxication. Our report highlights an elderly patient who developed encephalopathy and overt myoclonus after nasopharyngectomy secondary to intoxication by the components of the BIPP gauze. The patient’s impaired renal function, the amount of BIPP packing and the extensive nature of his wound likely predisposed him to BIPP toxicity. The myoclonus and delirium resolved promptly after removal of the BIPP packs. Clinicians should be aware of the clinical features of BIPP intoxication because of its common usage.

## Introduction

Bismuth iodoform paraffin paste (BIPP) gauze is an antiseptic dressing [1] routinely used in maxillectomy, nasopharyngectomy, and the management of epistaxis [2]. However, it should be remembered that BIPP can cause toxicity. Our report aims to highlight the risks of BIPP intoxication by discussing a patient with BIPP encephalopathy manifesting as overt myoclonus, recorded on videography.

## Case presentation

In May 2016, a 74-year-old ethnically Chinese man with comorbidities of renal impairment (baseline serum creatinine 151-203 µmol/L, creatinine clearance ~28-38 mL/min), hypertension, hyperlipidemia, diabetes mellitus, ischemic heart disease, and a previous left basal ganglia hemorrhage, underwent endoscopic nasopharyngectomy for recurrent nasopharyngeal carcinoma (NPC). He had been diagnosed with Stage III (T3N0M0, American Joint Committee on Cancer 7th Edition) NPC and completed chemoradiotherapy in 2014. In 2016, he was found to have a T1N0M0 recurrence in the left fossa of Rosenmüller of the nasopharynx. Endoscopic nasopharyngectomy with curative intent was performed in May 2016. This entailed a posterior septectomy, left inferior turbinectomy, mega maxillary antrostomy, and the drilling of the medial pterygoid plate followed by the resection of the cartilaginous left Eustachian tube en-bloc with the tumor in the left fossa of Rosenmüller. The resection cavity was reconstructed with a right-sided nasoseptal flap. The nasal cavity and nasopharynx were packed with five slips of BIPP gauze (impregnated with 20% bismuth subnitrate, 40% iodoform, and 40% liquid paraffin, Aurum Pharmaceuticals Ltd, Romford, United Kingdom). The resection margins were clear on the formalin-fixed paraffin-embedded specimens.

**Figure 1:**
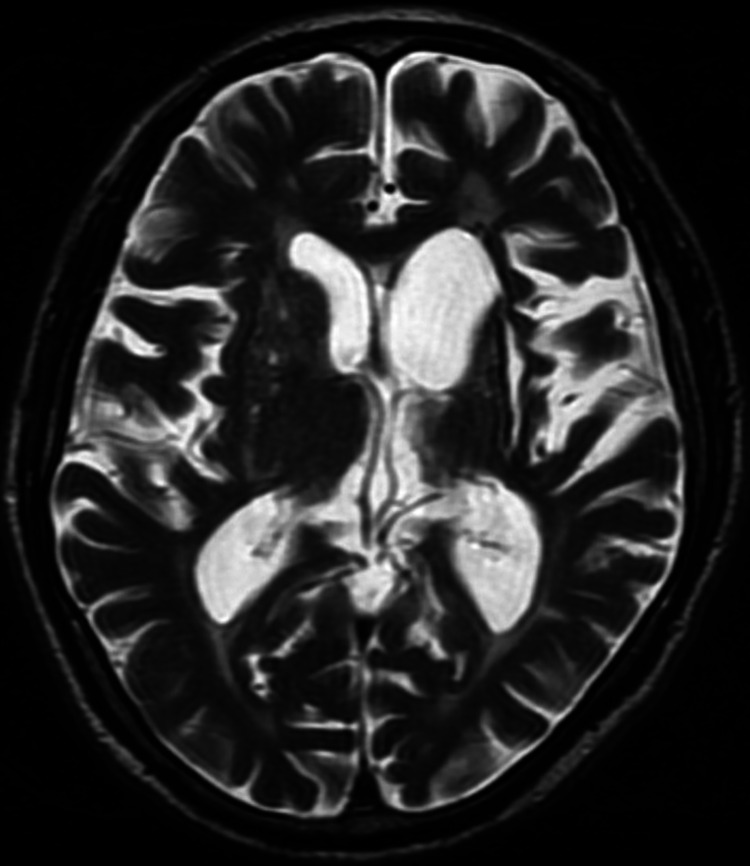
Magnetic resonance imaging (MRI) brain - a slice of the T2 sequence showing stable gliosis in the left basal ganglia with ex-vacuo dilatation as well as old infarcts in the right basal ganglia

**Figure 2:**
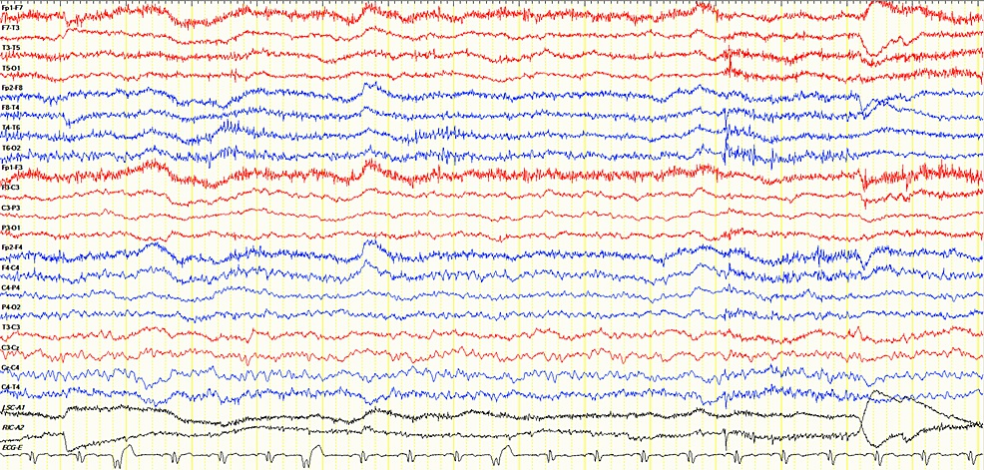
Electroencephalogram (EEG) - longitudinal bipolar montage (7 µV/mm sensitivity) shows continuous generalized slowing with the absence of a normal background rhythm

On postoperative day (POD) 6, the patient’s mental state began to fluctuate between agitation and drowsiness. He exhibited labile emotions and developed negative myoclonus in the form of asterixis in his upper limbs (see Video 1). There was no evidence of bleeding, sepsis, or airway obstruction. A review of his medications showed that he only required tramadol 50mg once a day on average for analgesia from POD1 to POD6. Further investigation of his delirium showed raised liver enzymes, predominantly affecting aspartate aminotransferase (183 U/L) and alanine aminotransferase (264 U/L) but also affecting gamma-glutamyl transferase (180 U/L) and alkaline phosphatase (139 U/L), with normal bilirubin levels (13 µmol/L). Sonography of the hepatobiliary system revealed no evidence of cirrhosis or liver metastasis. Lumbar puncture was not suggestive of meningitis. Computed tomography (CT) and magnetic resonance imaging (MRI) of the brain revealed only old infarcts on a background of microvascular ischemia and age-related involutional changes. These findings are best appreciated on the MRI brain (see Figure 1). Electroencephalogram (EEG) showed severe diffuse encephalopathy (see Figure 2).

**Video 1:** Negative myoclonus is demonstrated in the upper limbs with the patient unable to sustain wrist extension in his outstretched arms, resulting in brief, shock-like, involuntary movements

Intoxication by either iodine or bismuth in the BIPP packs was suspected, and the BIPP packs were removed on POD7. Mentation gradually improved over the next two weeks without further intervention. The transaminitis decreased. On POD7, urinary iodine level was measured to be 37,094 µg/L (range of normality 26-705 µg/L), suggesting high systemic absorption of the BIPP components. It dropped to 457 µg/L on POD26. Blood bismuth level was not obtained in the early postoperative period but was normal at 13.6 ng/l on POD18. Thyroid function test on POD9 showed an elevated free T4, low total T3 but normal TSH. The thyroid function test was also normalized by POD26.

## Discussion

Pharmacology

BIPP gauze is a ribbon gauze infused with one part bismuth subnitrate, two parts iodoform, and one part of sterilized liquid paraffin by weight [3].

Bismuth compounds have multiple routes of administration, most often being prescribed per-orally in anti-diarrheal medications, and also often used topically in gauzes such as BIPP. After bismuth enters the bloodstream, it crosses the blood-brain barrier to bind to enzymes involved in oxidative metabolism, leading to reduced oxygen consumption and reduced cerebral perfusion [4]. Bismuth is eliminated from the body unmetabolized via the renal and hepatobiliary routes, although the exact proportion contributed by each route is unknown [5].

Iodoform (CHI3) itself is lipid-soluble, hence it crosses the blood-brain barrier more easily than its metabolite iodine [6]. Upon contact with granulating tissue, iodoform releases iodine, therefore plasma iodine levels should reflect iodoform levels [6]. Over 90% of absorbed iodine is excreted in the urine [7], hence urinary iodine is an indicator of iodoform exposure.

Manifestations of BIPP toxicity

A well-studied outbreak of bismuth encephalopathy from oral bismuth in France and Australia in the 1970s [8] established the typical manifestations of oral bismuth toxicity to be prodromal depression, anxiety, irritability, and possibly mild incoordination. Deterioration then occurred rapidly over 24-48 hours with confusion, myoclonic jerks, dysarthria, and eventually coma. Recovery may be complete, or there may be mild residual memory loss.

Subsequently, there were reports of emerging cases of neurotoxicity from the use of BIPP impregnated gauze. Jones et al. [1] in 1990 reported malaise, insomnia, personality change, and limb rigidity in a 79-year-old man after BIPP packing of the bony cavity following excision of a mandibular keratocyst. Sharma et al. [9] in 1994 reported that restlessness, insomnia, confusion, and dysarthria developed in a 57-year-old lady after BIPP packing of dura mater in a wound post removal of large basal cell carcinoma. The patient recovered after the pack was removed. Ovaska et al. [10] in 2008 reported bismuth toxicity in a 67-year-old man after BIPP packing of a debrided sacral wound following resection of sacral chondroma. The patient developed confusion, drowsiness, and eventually myoclonic jerks [10]. Youngman et al. [11] in 2004 reported acute confusion, dysphonia, and gait dyspraxia in an 81-year-old man who received BIPP nasal packing for refractory epistaxis.

In these cases, BIPP was placed in close proximity to exposed vascular, visceral, or mucosal surfaces where direct absorption of BIPP into the bloodstream could have led to toxicity. It has also been proposed that when BIPP is placed near neuronal plexi, retrograde transportation of bismuth along the neurons towards the central nervous system may occur [10]. Another proposed mechanism of toxicity is the absorption of bismuth-laden saliva via the gastrointestinal route [5]. It is theorized that intestinal micro-organisms may convert bismuth to its soluble form and contributes to its toxicity [8].

Similar to bismuth, iodoform and iodine toxicity manifests with neuropsychiatric symptoms of malaise, decreased appetite, headache, agitation, depression or delirium [12], drowsiness and semi-coma [13], gastrointestinal symptoms of nausea and vomiting [14], and constitutional symptoms such as fever and tachycardia [12]. Additional findings of toxicity associated with iodine but not iodoform include dermatological eruptions, and laboratory findings of metabolic acidosis, renal failure, neutropenia [12], thyroid dysfunction [15], and liver dysfunction [16]. It is difficult to distinguish the symptoms of intoxication caused directly by iodoform from those caused by the released iodine, as a measurement of blood iodoform concentration level is not possible [12,17]. However, based on pharmacology, high levels of released iodine should suggest iodoform toxicity [12], and concomitant toxicity from both can occur.

In summary, the clinical manifestations of BIPP toxicity are listed in Table 1. Intoxication by the two components of BIPP, iodoform, and bismuth, shares similar neuropsychiatric symptoms of delirium, depression, and coma [12]. Myoclonus, however, is associated with bismuth [18] rather than iodine or iodoform intoxication.

**Table 1:** Symptoms of iodine/iodoform and bismuth intoxication

	Iodine/Iodoform intoxication	Bismuth intoxication
Neuropsychiatric	Malaise, decreased appetite, headache, agitation, depression or delirium, drowsiness, coma [12]	Malaise, insomnia, personality change [1], ataxia [19], dysphonia [11], dysarthria [9], gait dyspraxia [11], myoclonic jerks [10], delirium, drowsiness [10], coma [8]
Gastrointestinal	Nausea, vomiting [14, 15]	
Constitutional	Fever, tachycardia [12]	
Dermatologic	Skin iodine eruptions (in iodine toxicity) [12]	

Case discussion

In our patient, neurotoxicity developed after BIPP nasal packing. Following a period of delirium, characteristic myoclonus was seen in the distal upper limbs (see Video 1), the most common site of bismuth-induced myoclonus [18]. Upon removal, the patient demonstrated characteristic reversibility of symptoms [11] with no residual neurological complications. The clinical symptoms and EEG findings were consistent with that of bismuth encephalopathy, even though blood bismuth levels were not obtained at the beginning of intoxication.

Our patient’s altered mental status may also have been aggravated by concomitant iodoform or iodine toxicity from the BIPP packing, as urinary iodine levels were high during the period of symptoms. Iodoform and iodine toxicity likely contributed to the patient’s agitation, drowsiness, and delirium. Iodine toxicity may additionally have caused transient thyroid dysfunction and liver enzyme derangement.

The patient’s timing of neurological recovery was consistent with the expected time of two weeks to one month following iodoform intoxication [13]. Recovery time following bismuth intoxication is less well established, although rapid improvement has been seen in as fast as two days [20].

In our patient, there were multiple risk factors for intoxication. First, the placement of packing in the nasal and nasopharyngeal cavity allowed bismuth and iodine to enter the gastrointestinal tract via a postnasal drip. Second, the large size of the wound cavity and injured mucous membranes of the nasopharynx and nasal cavity facilitated direct absorption of bismuth and iodine into the bloodstream. Third, the venous drainage of the nasal cavity communicates with the cavernous sinus intracranially through the pterygoid venous plexus, potentially increasing the risk of neurological side effects. Last, renal impairment placed the patient at risk of both bismuth and iodoform toxicity as both substances are excreted renally [5,12,17].

## Conclusions

Popularized in the First World War, BIPP gauze is still being used frequently by maxillofacial surgeons and otolaryngologists worldwide. However, delirium, encephalopathy, and coma can develop from the use of BIPP gauze. To decrease the risk of intoxication, surgeons should take into account the patient’s renal and hepatic function, the size and condition of the wound, and the quantity of packing. Familiarity with the manifestations of BIPP intoxication is necessary for clinicians to promptly diagnose and effectively treat it. Alternative packing materials should also be sought. Since this patient encountered toxicity, we have replaced the BIPP pack with ribbon gauze coated with tetracycline ointment for nasal packing. Flavin-soaked ribbon gauze can be another alternative.
